# Predicting perinatal mortality based on maternal health status and health insurance service using homogeneous ensemble machine learning methods

**DOI:** 10.1186/s12911-022-02084-1

**Published:** 2022-12-28

**Authors:** Dawit S. Bogale, Tesfamariam M. Abuhay, Belayneh E. Dejene

**Affiliations:** College of Informatics, University of Gondar, Gondar, Ethiopia

**Keywords:** Homogenous ensembles, Machine learning, Perinatal mortality, Maternal health, Health insurance

## Abstract

**Background:**

Perinatal mortality in Ethiopia is the highest in Africa, with 68 per 1000 pregnancies intrapartum deaths. It is mainly associated with home delivery, which contributes to more than 75% of perinatal deaths. Financial constraints significantly impact timely access to maternal health care. Financial incentives, such as health insurance, may address the demand- and supply-side factors. This study, hence, aims to predict perinatal mortality based on maternal health status and health insurance service using homogeneous ensemble machine learning methods.

**Methods:**

The data was collected from the Ethiopian demographic health survey from 2011 to 2019 G.C. The data were pre-processed to get quality data that are suitable for the homogenous ensemble machine-learning algorithms to develop a model that predicts perinatal mortality. We have applied filter (chi-square and mutual information) and wrapper (sequential forward and sequential backward) feature selection methods. After selecting all the relevant features, we developed a predictive model using cat boost, random forest, and gradient boosting algorithms and evaluated the model using both objective (accuracy, precision, recall, F1_score, ROC) and subjective (domain expert) based evaluation techniques.

**Results:**

Perinatal mortality prediction models were developed using random forest, gradient boosting, and cat boost algorithms with the overall accuracy of 89.95%, 90.24%, and 82%, respectively. Risk factors of perinatal mortality were identified using feature importance analysis and relevant rules were extracted using the best performing model.

**Conclusions:**

A prediction model that was developed using gradient boosting algorithms was selected for further use in the risk factor analysis, generating relevant rules, development of artifacts, and model deployment because it has registered better performance with 90.24% accuracy. The most determinant risk factors of perinatal mortality were identified using feature importance and some of them are community-based health insurance, mother's educational level, region and place of residence, age, wealth status, birth interval, preterm, smoking cigarette, anemia level, hemoglobin level, and marital status.

**Supplementary Information:**

The online version contains supplementary material available at 10.1186/s12911-022-02084-1.

## Background

Perinatal mortality refers to a fatal death at or after 28 weeks of pregnancy (stillbirth) and includes death within 7 days of life after birth [1, 2]. According to the World Health Organization (WHO) 2019 report, there were 2.6 million newborn infants globally, but more than 8200 died within a day [3]. Among the 133 million newborn infants alive each year, 2.8 million died in the first week of life after birth/at birth, and the majority occurred in low-income level countries [3]. Given the fast approaching deadlines for reaching the Millennium Development Goals, the international community supports low- and middle-level income countries to renew their commitment towards reducing maternal and infant mortality rates by improving access to maternal, neonatal, and perinatal health services [4].

Perinatal mortality in Ethiopia is the highest in Africa, with 68 per 1000 pregnancies and Intrapartum deaths [5]. Ethiopia shares and values the Sustainable Development Goals and has been trying to achieve the target of reducing neonatal mortality to below 12 per 1000 live births by 2030 [6]. However, the reduction of neonatal, infant and under-five mortalities cannot be realized without a substantial reduction of perinatal mortality [7]. It is mostly associated with home deliveries, which contributed to more than 75% of all perinatal deaths due to the lack of awareness about health insurance services during birth, and it continued to be an essential part of the third sustainable development goal which aims to end preventable children's deaths by 2030 [6].

Financial constraints have a significant impact on timely access to maternal health care, such as Antenatal Care (ANC), skilled care at delivery, access to facility-based deliveries, postnatal care, and perinatal [8]. Over 100 million individuals pay out-of-pocket payments to get health treatments that have proven difficult to obtain for millions of poor people, resulting in increased morbidity and mortality [9]. WHO recommends community-based health insurance as one of the approaches for reducing pay out-of-pocket expenditures for registered families which, in turn, reduces morbidity and mortality [10]. The association of community-based health insurance with reduced maternal and infant mortality was apparent but it is impossible to reduce the infant mortality rate, without reducing the perinatal mortality [8]. Financial incentives, such as health insurance, can address the demand- and supply-side factors that may impact maternal, neonatal, and perinatal health results [11]. To this end, the Ethiopian Ministry of Health has been working for years to make health services accessible for women through community and facility-based interventions to increase the survival of newborns and children [6]. Despite these interventions, perinatal death remains an issue in Ethiopia, in particular; home delivery remains the challenge to reduce perinatal mortality [11]. Still, 74% of women give birth outside health institutions without skilled care attendants in Ethiopia [5, 12, 13].

Developing perinatal mortality prediction model using machine learning algorithms facilitates preventive actions. Machine learning, which aims to teach computers to perform what naturally involves humans learning from experience [5], includes supervised learning, unsupervised learning, and reinforcement learning [5]. Machine learning allows processing of large healthcare datasets and extracting clinical insights that support clinicians in planning and giving care, resulting in better outcomes, lower healthcare costs, and increased patient satisfaction [14].

This study, hence, aims at predicting perinatal mortality based on maternal health status and health insurance service using homogeneous ensemble machine learning methods by investigating the following research questions (1) what is the underline structure and evolution of perinatal mortality in Ethiopia over time? (2) Which homogeneous ensemble machine learning method is suitable to predict perinatal mortality in Ethiopia effectively? (3) What are the determinant factors of perinatal mortality in Ethiopia? (4) What are the important rules that may shape strategies, policies, and interventions toward preventing and/or reducing perinatal mortality in Ethiopia?

The rest of this paper is organized as follows: section two presents related works, section three discusses materials and methods used, section four mentions experimental setup, result, and discussion, and section five presents conclusion.

## Related work

Several studies investigated perinatal mortality in Ethiopia using different methods. Getachew et al. [14] investigated perinatal mortality and associated risk factors using a case–control study between 2008 and 2010 using a total of 1356 newborns’ data (452 cases and 904 controls). Subgroup binary logistic regression analyses was done to identify associated risk factors for perinatal mortality, stillbirths, and early neonatal deaths. The study reported that the perinatal mortality rate was 85/1000, and after or at 28 weeks of birth death accounts for 87% [14]. Adjusted odds ratios revealed that obstructed labor, malpresentation, preterm birth, death during delivery, hemorrhage, and hypertensive disorders of pregnancy was independent predictor for high perinatal mortality.

Another study was conducted by Yemisrach et al. [15] on factors associated with perinatal mortality among public health deliveries in Addis Ababa, Ethiopia using an unmatched case–control study and secondary data that was collected between 1^st^ January to 30^th^ February 2015. In this study, a total of 1113 (376 cases and 737 controls) maternal charts were reviewed and the mean age of the mothers for cases and controls were 26.47 ± 4.87 and 26.95 ± 4.68, respectively. Five hundred ninety-seven (53.6%) mothers delivered for the first time and factors that are significantly associated with increased risk of perinatal mortality were birth interval less than 2 years, preterm delivery, anemia, congenital anomaly, previous history of early neonatal death, and low birth weight. This study also reported that the use of a partograph was also associated with decreased risk of perinatal mortality. Bekele et al. [16] studied the effect of community-based health insurance on the utilization of outpatient health care services in Yirgalem town, Southern Ethiopia. This study used both quantitative and qualitative (mixed) research approaches using a comparative cross-sectional study design. A randomly selected sample of 405 (135 members and 270 non-members) household heads was used for quantitative analysis. Multivariate logistic regression was employed to identify the effect of community-based health insurance on healthcare utilization. This study revealed that members of households with community-based health insurance were about three times more likely to utilize outpatient care than their non-member counterparts [AOR: 2931; 95% CI (1.039, 7.929); p-value = 0.042]. Finally, the researchers conclude that community-based health insurance is an effective tool to increase the utilization of healthcare services and provide the scheme to member households. Bitew et al. [17] employed machine learning approach for predicting under-five mortality in Ethiopia using data from the 2016 Ethiopian Demographic and Health Survey. In in this study, a total of 624 data were used to develop a prediction model using random forests, logistic regression, and K-nearest neighbors and registered an accuracy of 67.2%, 59.9% and 46.3%, respectively.

However, the aforementioned studies focused on identifying determinant risk factors only with a small dataset that cover limited geographical areas. Besides, these studies did not develop a predictive model, did not design an artifact that can be used by potential users, and did not generate rules that allow the development of evidence-based preventive strategies, policies, and interventions. On the other hand, machine learning algorithms have proven to be effective and efficient in predicting child mortality in African countries such as South Africa [18] and Uganda [19]. This study, hence, is motivated to fill these gaps by constructing a predictive model, identifying risk factors, designing artifacts, and generating relevant rules that help to develop evidence-based strategies, policies and interventions towards preventing, controlling and/or ending perinatal mortality in Ethiopia. Emmanuel et al. [20] explored application of machine learning methods for predicting infant mortality in Rwanda using Rwanda demographic health survey 2014–15 dataset and showed effectiveness and explain ability of machine learning algorithms such as Random forest, decision tree, support vector machine and logistic regression. For this reason, ensemble machine learning algorithms were selected for experiment in this study.

## Materials and methods

**Fig. 1:**
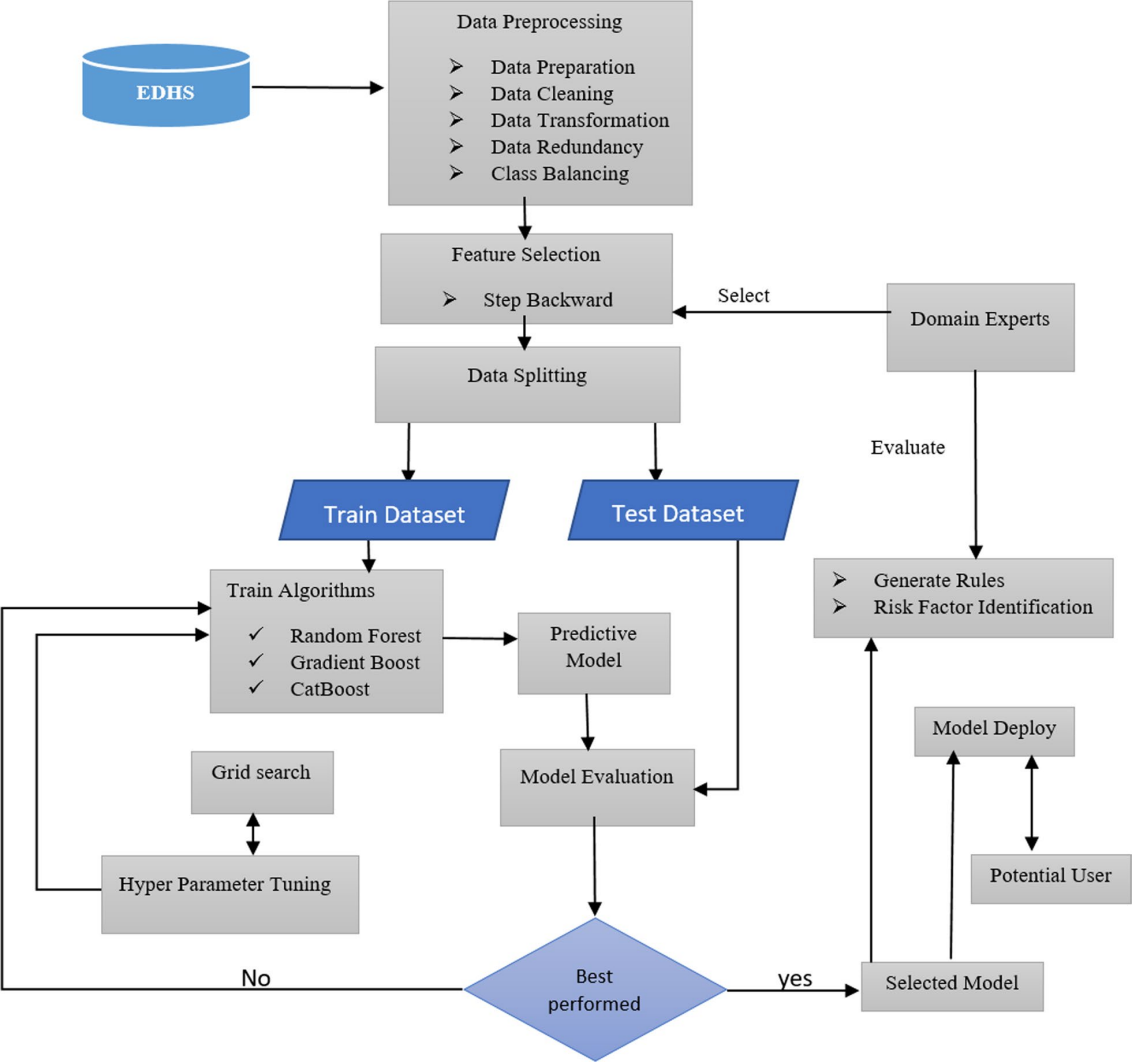
The proposed model architecture

Figure 1 depicts methodological flow chart that was implemented in this study to construct a perinatal mortality prediction model, identify risk factors, extract relevant rules, and design artifacts.

### Data collection and preprocessing

In this study, a secondary data from the Ethiopia Demographic and Health Surveys (EDHS) which was collected by the Ethiopian Central Statistical Agency in 2011, 2016, and 2019 G.C, in five years intervals were used. The EDHS is a nationally representative household survey that collects data about demography, health, and nutrition for the purpose of impact monitoring and evaluation. To conduct this study, we have extracted data related to perinatal mortality and maternal determinants. The raw data contains a total of 45 attributes (perinatal mortality status as a target) and 109,531 instances before applying any feature selection techniques. The data imputation method (mode for categorical data and mean for continuous data) was employed to substitute the missing values. Outliers were identified using a boxplot and replaced using the Interquartile Range (IQR) scores. Data discretization was applied to transform some of the features. For example, the feature ‘education level of mothers (v106)’ has 8 different values which were transformed into five different values (illiterate (1), grade 1–8 (elementary), grade9-12 (secondary), grade 12+ (tertiary), and higher education (university and college)). We have conducted four feature selection experiments to select the most relevant predictors or features using filter (mutual information and chi-square) and wrapper (sequential forward and sequential backward) methods. As a result, the sequential backward feature selection method has registered the highest performance with 90.5% of accuracy and produced 13 important features, see Table 1. The baseline fitness improvement (BFI) is used to limit the amount/number of features to be selected using different feature selection methods [21]. Besides, the domain experts who work at the University of Gondar comprehensive specialized hospital recommended 4 additional features namely preterm, the highest educational level, visited health facility in last 12 weeks, and birth interval. Finally, the total of 17 features, selected by both objective and subjective methods, were used for further analysis, see Table 1. The synthetic minority over-sampling technique (SMOTE) was implemented to handle the class imbalance in the training dataset. The main reason that we used SMOTE is it avoids loss of valuable information [22, 23]. Before applying SMOTE, the data contains 109,531 instances (74,770 alive, and 34,761 died), whereas after applying SMOTE, the data account for 148,659 instances (74,329 alive and 74,330 died), and we used the balanced dataset to develop the final predictive model.

**Table 1:** Features selected by sequential-backward feature selection and domain experts

No	Chi_best	Mi_best	SFFS	SBFS
**Features Selected by Feature Selection Methods**				
1	Maternal age	Maternal age	ever-married sample	Community health insurance
2	Literacy	Preterm	currently pregnant	Smokes cigarettes
3	frequency of reading newspapers or magazine	highest educational level	current contraceptive method	Region
4	Family size	Religion	current use by method type	Type of cooking fuel
5	Wealth index	Ethnicity	heard family planning on radio last few months	Wealth index
6	Preterm	Family size	heard family planning on TV last few months	Current contraceptive method
7	current contraceptive method	frequency of listening to the radio	heard family planning in newspaper/magazine last few months	Hemoglobin level
8	current use by method type	type of cooking fuel	visited health facility last 12 months	Occupation
9	visited health facility last 12 months	wanted last children	Smokes pipe full of tobacco (women)	Maternal age
10	currently breastfeeding	currently breastfeeding	Chews tobacco	Marital status
11	when children put to the breast	Hemoglobin level	smokes other	Anemia level
12	Chews tobacco	anemia level	Community health insurance	Chews tobacco
13	smokes other nicotine	Marital status	Health insurance type: provided by the employer (women)	Place of residence
Accuracy	84.43	85.32	85.5	90.5
**Features Selected by Domain Experts**				
14				Preterm
15				Highest educational level
16				Visited health facility last 12 weeks
17				Birth interval

### Model development methods

To conduct this study, a design science research approach was implemented and a secondary data that was extracted from the 2011, 2016 and 2019 Ethiopian Health Demographic Survey was employed. We have conducted all the experiments using Python, a general-purpose programming language. Different data pre-processing techniques such as handling missing values for categorical data using the mode imputation method, handling data transformation using binning, discretization, and normalization according to the data type, and handling class imbalance using SMOTE were employed. Several experiments were conducted using different feature selection methods such as filter (Mutual information and Chi-square), wrapper (step forward and step backward) and domain experts. And 17 features that were selected by step backward feature selection methods and features that were recommended by domain experts were used to develop the classification model. To develop the predictive model, we have implemented homogeneous ensemble machine learning algorithms such as Random Forest, CatBoost, and gradient Boost, and the hyper parameters of each algorithm were tuned using grid search. We have selected those three homogeneous ensemble machine learning algorithms due to their explain ability, strengths of preventing bias and over fittings, and nature of the data. Finally, the performance of the predictive models was evaluated using objective (accuracy, precision, recall, f1_score, ROC, and K-fold cross-validation (k = 10)) and subjective (domain experts’ opinion) methods. The best performing model was used to identify risk factors using feature importance, generate relevant rules for decision making, and develop an artifact that can be used by potential users such as health care professionals and parents.

## Results and discussion

In this section, the research questions that were raised at the introduction section of this study are discussed sequentially.

### What is the underline structure and evolution of perinatal mortality in Ethiopia over time?

**Fig. 2:**
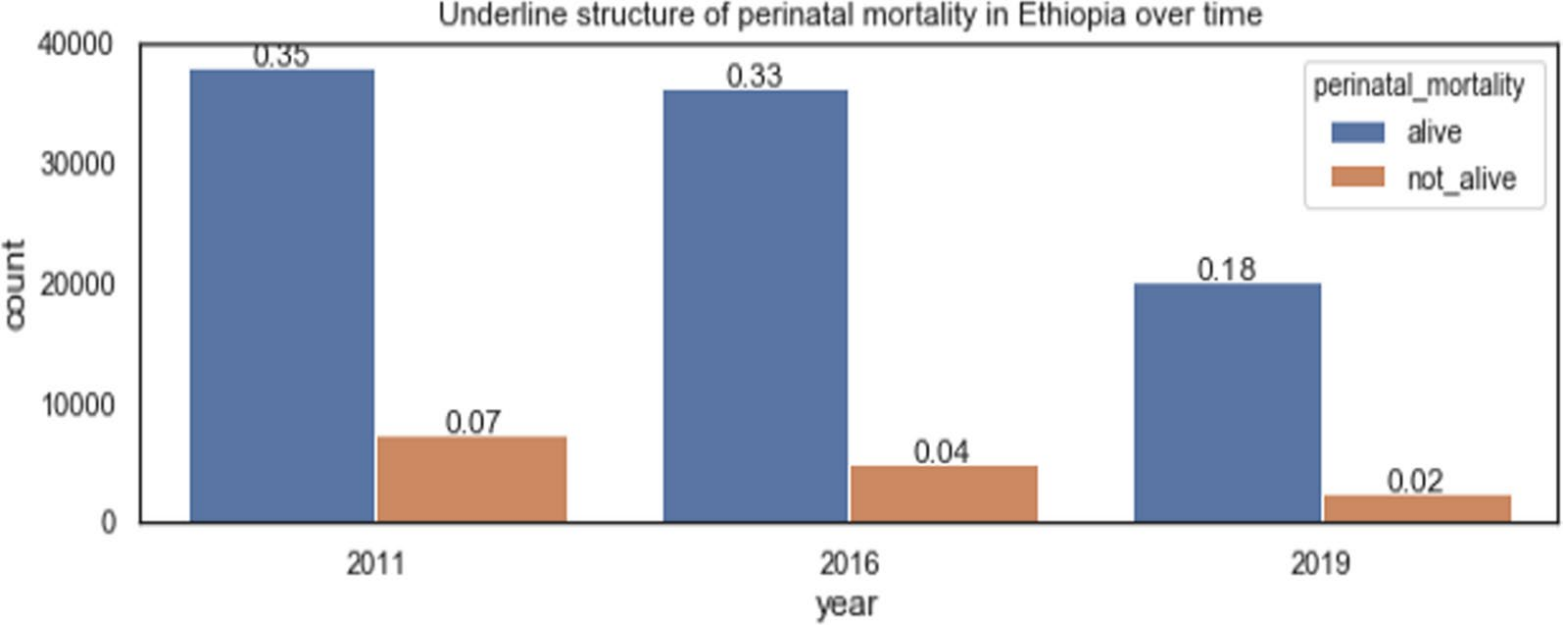
Perinatal mortality over time in Ethiopia

Perinatal mortality has been reducing over time in Ethiopia. This is because of the increase in the number of hospitals, especially in rural areas, and the introduction of community-based health insurance which encourages pregnant women to visit the hospital to give birth. But, due to the COVID-19 pandemic, the data collected in 2019 were twice as smaller as in previous years, as shown in Fig. 2.

**Fig. 3:**
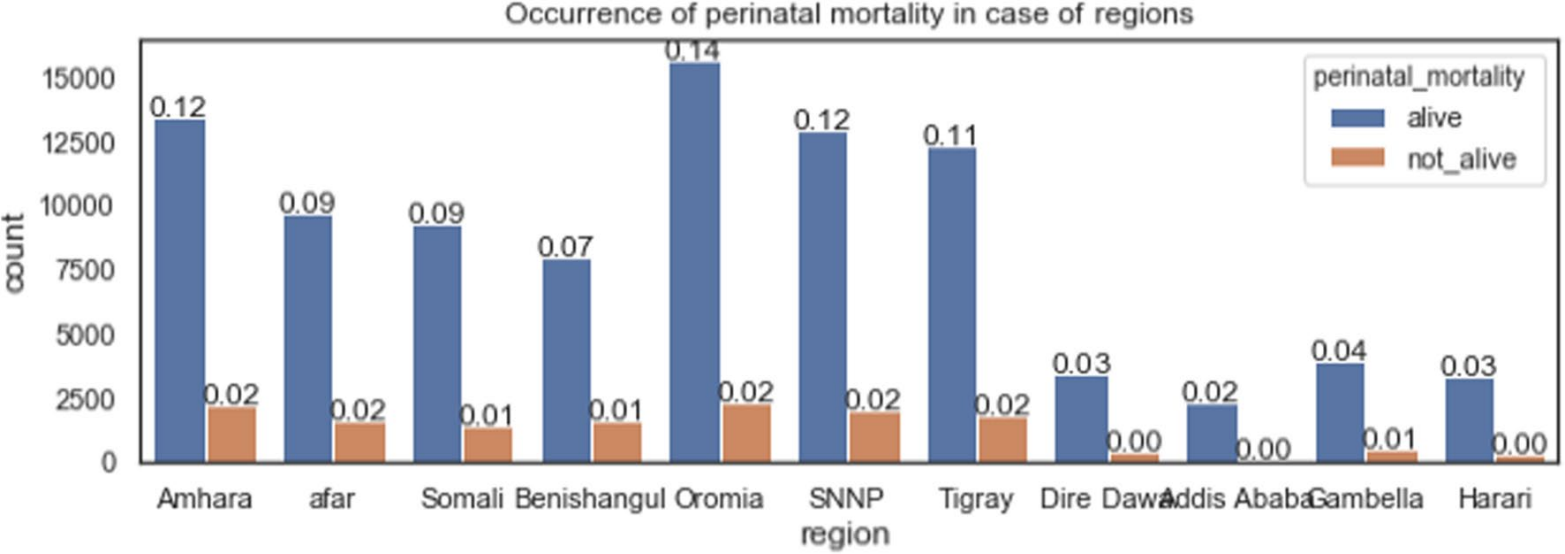
The perinatal mortality across the regions in Ethiopia

The perinatal mortality across the regions of Ethiopia was also investigated. Among nine regions and two city administrations of Ethiopia, Amhara and Oromia regions registered higher perinatal mortality compared to other regions, as shown in Fig. 3.

**Fig. 4:**
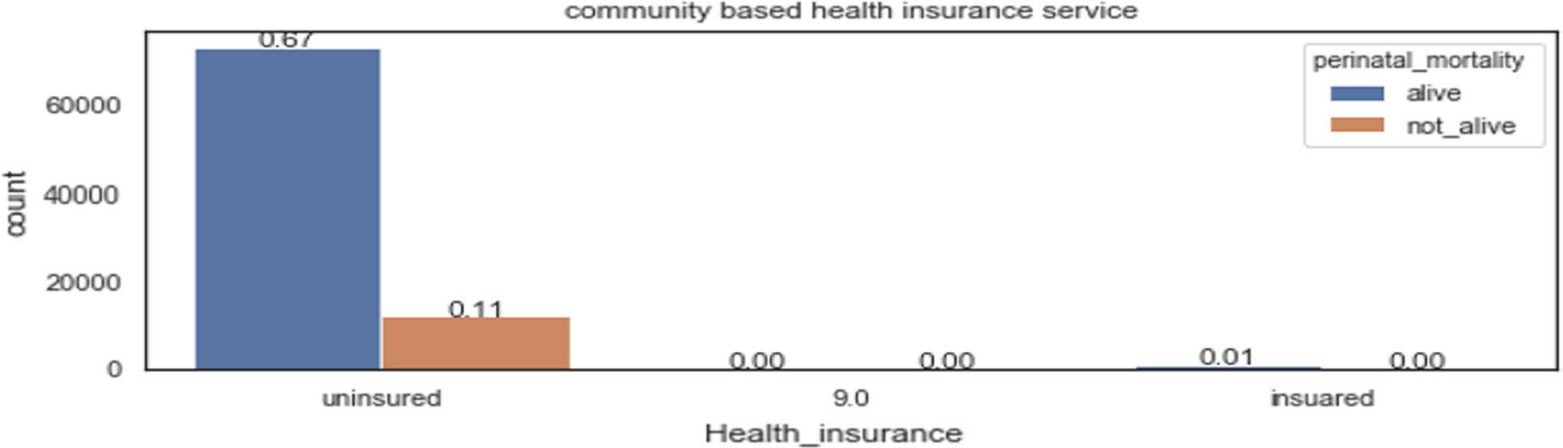
perinatal mortality compared to health insurance

Having health insurance service leads to almost zero perinatal mortality because it helps mothers to get affordable access to a health facility in time, as shown in Fig. 4.

### Which homogeneous ensemble machine learning algorithm predicts perinatal mortality in Ethiopia effectively?

Three experiments were conducted to build a perinatal mortality prediction model using classification algorithms namely; Gradient Boost, CatBoost, and random forest classifiers. A tenfold cross-validation method was implemented during the training. To select an optimal model with better performance, grid search was used to tune the best parameters of each classification algorithm. The values for parameters of each homogeneous ensemble machine learning algorithm are identified by Grid search for all experiments.

As a result, gradient boosting with the aforementioned, see Table 2, parameters performed better with 99.72% recall, 90.24% accuracy, 92.80% f1-score, 86.96% ROC and 87.24% precision. The recall indicates that there is a maximized true positive rate and a minimized false-negative rate meaning; there is a minimum false-negative rate. The confusion matrix of Gradient Boosting, Cat Boost and Random Forest algorithms is presented in Table 3.

**Table 2:** Tuned parameters using grid-search

Algorithm	Parameter	Values
Gradient boost	Criterion	friedman_mse
	max_depth	15
	n_estimators	100
	Random_state	42
Random forest	Criterion	Entropy
	max_features	sqrt
	min_samples_split	3
	n_estimators	200
	random_state	0
	max_depth	20
	max_leaf_nodes	400
	n_jobs	− 1
Cat boost	Verbose	False
	eval_metric	AUC
	Iterations	500
	thread_count	None
	random_state	1.0

**Table 3:** Confusion matrix

Gradient boosting		Predicted class	
		Died	Alive
*Actual class*	Died	8125	2736
	Alive	167	18,704

Cat boost		Predicted class	
		Died	Alive
Actual class	Died	7056	3764
	Alive	1750	17,162

Random forest		Predicted class	
		Died	Alive
*Actual class*	Died	8033	2902
	Alive	36	18,711

**Fig. 5:**
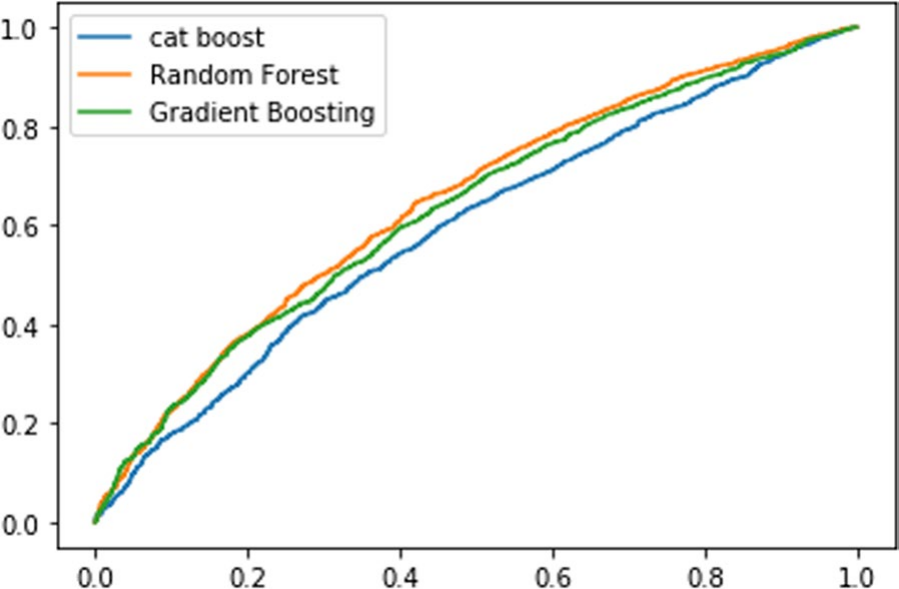
ROC AUC curve

By using all the tuned parameters (see Table 2), we have developed a predictive model using gradient boosting, cat boost, and random forest algorithms. Based on the tuned parameters, we have also evaluated the developed predictive model using different evaluation metrics, and the gradient boost algorithm is selected as the best homogenous ensemble machine learning algorithm for predicting perinatal mortality based on maternal health status and health insurance service in Ethiopia. The overall results of each experiment are summarized in Table 4 and Fig. 5.

**Table 4:** Overall performance of models

Evaluation	Algorithms		
	Gradient boost (%)	Cat boost (%)	Random forest (%)
Accuracy	**90.24**	81.45	89.95
Precision	87.24	82.01	86.42
Recall	99.72	90.75	99.54
ROC	96.96	87.98	96.50
F1_Score	92.80	86.16	92.72
K-fold cross-validation for k = 10	94.72	88.26	90.78
Specificity	74.81%	82.01%	86.57%

Bold indicates the algorithm that registered high performance

### What are the determinant factors of perinatal mortality in Ethiopia?

Feature importance analysis was conducted to identify risk factors of perinatal mortality in Ethiopia using the best-performing model which was developed using gradient boosting. As a result, factors that are significantly associated with perinatal mortality in Ethiopia are the birth interval of less than 2 years, region of residence, maternal age, wealth index, occupation, anemia level, health facility visit in the last 12 weeks, marital status, current contraceptive use, type of cooking fuel, education level, preterm delivery, hemoglobin level, place of residence, community health insurance, smoking cigarette, and chewing tobacco, see Table 5. Besides, community/mutual health insurance is one of the risk factors that are associated with perinatal mortality in Ethiopia. We have also conducted the feature importance analysis using the models that were developed using Cat Boost and Random Forest algorithms and the results are presented in Additional file 2: Appendix II.

**Table 5:** Risk factors that were identified using feature importance analysis

No	Feature code	Feature description	Feature importance value
1	Bord	Birth interval	0.291119
2	V024	Region	0.122834
3	V013	Maternal age	0.077887
4	V190	Wealth index	0.072292
5	V717	Maternal occupation	0.055206
6	V457	Anemia level	0.054080
7	V394	Visited health facility last 12 week	0.038728
8	V501	Marital status	0.030207
9	V312	Current contraceptive	0.026625
10	V161	Types of cooking fuel	0.026059
11	V106	Educational level	0.021210
12	V228	Preterm	0.019435
13	V455	Hemoglobin level	0.016383
14	V025	Place of residence	0.009877
15	V481a	Community/mutual health insurance	0.007053
16	V463a	Smoke cigarettes	0.006037
17	V463c	Chews tobacco	0.002598

### What are the important rules that may shape strategies, policies, and interventions toward reducing and/or preventing perinatal mortality in Ethiopia?

**Fig. 6:**
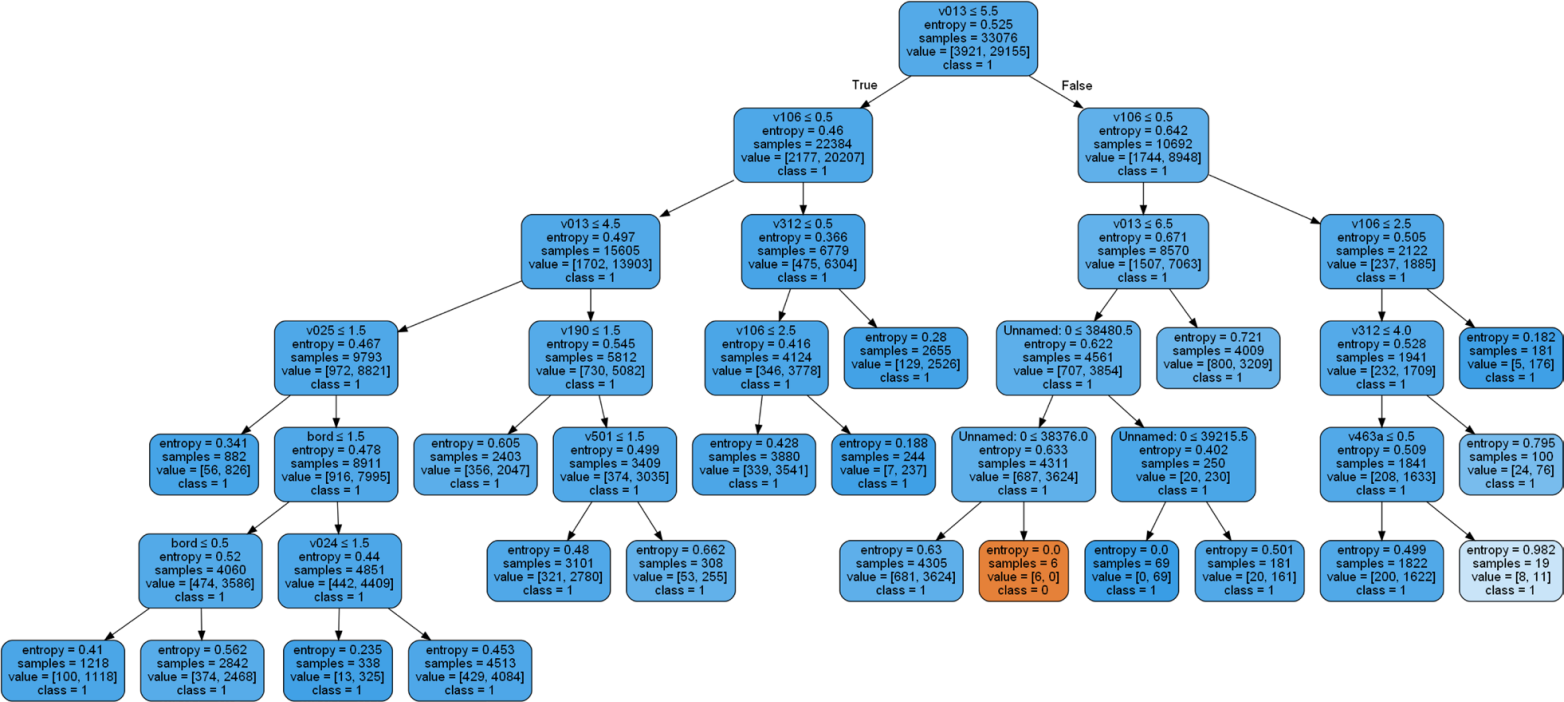
Generated rule by decision tree

The most relevant rules were generated from the best-performed algorithm (gradient boost) model, and the rules were validated by the domain experts who works at the University of Gondar comprehensive specialized hospital. Sample rules are presented in Additional file 1: Appendix I and Fig. 6 presents a decision tree of relevant rules that were generated by the best-performing algorithm.

## Discussion

Predicting perinatal mortality plays an important role to prevent and/or control child mortality in Ethiopia and maintain the health of future generations. This study, hence, targets to investigate the underlying structure of perinatal mortality, develop a machine learning model that predicts perinatal mortality, identify risk factors of perinatal mortality in Ethiopia and recommend relevant rules that help different stakeholders to make an evidence-based decision towards combating perinatal mortality in Ethiopia. To this end, data were collected from the Ethiopian demographic health survey and pre-processed using different methods. The explanatory analysis shows that perinatal mortality has been reducing over time in Ethiopia due to an increase in the number of hospitals in rural areas and the introduction of community-based health insurance which encourages pregnant women to visit the hospital to give birth. The proposed model, which was developed using a gradient boosting machine learning algorithm, achieved an overall performance of 90.24%, which is a better result compared to a result achieved by previous studies [17, 20, 24] which achieved 83% overall performance using gradient boosting machine learning algorithm. Based on the feature importance analysis, perinatal mortality in Ethiopia is significantly associated with health insurance and maternal health status such as birth interval of less than 2 years, preterm delivery, anemia, congenital anomaly, educational status, family size, occupation, marital status, perceived quality of care, the first choice of place for treatment during illness, previous history of perinatal death, not receiving tetanus toxoid immunization, and lack of iron supplementation. Besides, relevant rules, that facilitate evidence-based decision, were generated using the best-performing algorithm. The proposed model was deployed on the cloud using Heroku and Flask framework and can be freely accessed by potential users via this link: http://perinatal-mortality.herokuapp.com/.

## Conclusion

Perinatal mortality refers to fatal death at or after 28 weeks of pregnancy (stillbirth) and includes death within 7 days of life after birth. Among the 7.7 million deaths of children aged below five years in 2018, 3.1 million were in their perinatal period within 28 weeks and the first week of life. This study, thus, aimed to develop a machine learning model that predicts perinatal mortality in Ethiopia using homogeneous ensemble machine learning methods. To this end, design science research approach was employed and the proposed model was constructed using homogeneous ensemble machine learning algorithms namely gradient boost, random forest, and cat boost. The gradient boost algorithm has registered the highest performance with 99.72% recall, 90.24% accuracy, 92.80% f1-score, 86.96% ROC and 87.24% precision. We have also identified the determinant risk factors by conducting a feature importance analysis on the best-performed algorithms and some of the most determinant risk factors were maternal residence, level of education, birth interval, and community-based health insurance. The most relevant rules, that help to formulate evidence-based strategies and policies towards preventing, controlling and/or ending perinatal mortality in Ethiopia, were generated from the best-performing model, and the rules were validated by the domain experts. In the future, additional and recent data will be collected and heterogeneous ensemble machine learning algorithms will be considered. The limitation of this study is we were not able to measure the effect of the model in the real-world environment.

## Supplementary Information

**Additional file 1.** Appendix I-relevant rules that may help policy makers in preventing and/or controlling perinatal mortality.

**Additional file 2.** Appendix II-feature importance results.

## Data Availability

The datasets generated and/or analyzed during the current study are available in the ‘perinatal_dataset-’ repository on GitHub: https://github.com/dawitemu1/perinatal_dataset.

## Abbreviations

ANC: Antenatal care
CBHI: Community-based health insurance
EDHS: Ethiopia demographic health survey
MH: Maternal health
OOP: Out-of-pocket
PNC: Postnatal care
ROC: Receiver operating characteristics
SDGs: Sustainable Development Goals
SMOTE: Synthetic minority over-sampling technique
WHO: World Health Organization
