# Supplementary material for: Predicting perinatal mortality based on maternal health status and health insurance service using homogeneous ensemble machine learning methods
Source: BMC Med Inform Decis Mak. 2022 Dec 28;22:341. doi: 10.1186/s12911-022-02084-1 (PMC9795949; doi:10.1186/s12911-022-02084-1)
Supplement: Supplementary file 1 — Additional file 1. Appendix I-relevant rules that may help policy makers in preventing and/or controlling perinatal mortality. [file 12911_2022_2084_MOESM1_ESM.docx]

**Appendix I-Relevant rules that may help policy makers in preventing and/or controlling Perinatal Mortality**

Rule 1:- IF currently breast feeding and preterm == 'no' AND maternal education== 'no education' AND wanted least children == 'wanted then' AND smoke ciggrate == 'no' AND health insurance provide by employer == 'no' AND smoke Tabaco == 'never in union' AND types of cooking fuel == 'wood' AND occupation == 'not working AND wealth index== 'poorest' AND maternal age == '35-39' AND place of residence == 'rural' AND Community based health insurance == 'no' AND Then child=='Alive'

Rule 2:- IF currently breast feeding and preterm == 'no' AND maternal education== 'no education' AND wanted least children == 'wanted then' AND smoke ciggrate == 'no' AND health insurance provide by employer == 'no' AND smoke Tabaco == 'never in union' AND types of cooking fuel == 'wood' AND occupation == 'not working AND wealth index== 'poorest' AND maternal age == '40-44' AND place of residence == 'urban' AND Community based health insurance == 'no' AND Then child=='Died'

Rule 3:- IF currently breast feeding and preterm == 'no' AND maternal education== 'no education' AND wanted least children == 'wanted then' AND smoke ciggrate == 'no' AND health insurance provide by employer == 'no' AND smoke Tabaco == 'never in union' AND types of cooking fuel == 'wood' AND occupation == 'not working AND wealth index== 'poorest' AND maternal age == '45-49' AND place of residence == 'rural' AND Community based health insurance == 'no' AND Then children=='Alive'

Rule 4:- IF currently breast feeding and preterm == 'no' AND maternal education== 'no education' AND wanted least children == 'wanted then' AND smoke ciggrate == 'no' AND health insurance provide by employer == 'no' AND smoke Tabaco == 'never in union' AND types of cooking fuel == 'wood' AND occupation == 'not working AND wealth index== 'poorest' AND maternal age == '45-49' AND place of residence == 'rural' AND Community based health insurance == 'no' AND Then children=='Alive'

Rule 5:- IF currently breast feeding and preterm == 'no' AND maternal education== 'no education' AND wanted least children == 'wanted then' AND smoke ciggrate == 'no' AND health insurance provide by employer == 'no' AND smoke Tabaco == 'never in union' AND types of cooking fuel == 'wood' AND occupation == 'not working AND wealth index== 'poorest' AND maternal age == '15-19' AND place of residence == 'rural' AND Community based health insurance == 'no' AND Then children== 'Died'
