# Supplementary material for: Predicting perinatal mortality based on maternal health status and health insurance service using homogeneous ensemble machine learning methods
Source: BMC Med Inform Decis Mak. 2022 Dec 28;22:341. doi: 10.1186/s12911-022-02084-1 (PMC9795949; doi:10.1186/s12911-022-02084-1)
Supplement: Supplementary file 2 — Additional file 2. Appendix II-feature importance results. [file 12911_2022_2084_MOESM2_ESM.docx]

**Appendix II Feature Importance Result**

1. **Cat Boost Algorithm Feature Importance Result**

| Feature | Feature importance values |
| --- | --- |
| v013 | 0.142902 |
| v024 | 0.1372867 |
| bord | 0.1061283 |
| v190 | 0.0805674 |
| v717 | 0.07692465 |
| v106 | 0.07649285 |
| v312 | 0.06843245 |
| v457 | 0.0550137 |
| v161 | 0.05342789 |
| v501 | 0.05080563 |
| v394 | 0.03886669 |
| v228 | 0.02917583 |
| v455 | 0.02677905 |
| v025 | 0.02645179 |
| v463a | 0.01056291 |
| v481a | 0.00905534 |
| v463c | 0.00363744 |

1. **Random Forest Algorithm Feature Importance Results**

| Feature | Feature importance values |
| --- | --- |
| v013 | 0.166632 |
| v024 | 0.123759 |
| bord | 0.121659 |
| v190 | 0.082136 |
| v106 | 0.069755 |
| v717 | 0.064254 |
| v312 | 0.057373 |
| v457 | 0.054248 |
| v161 | 0.052818 |
| v501 | 0.052229 |
| v394 | 0.035494 |
| v455 | 0.03323 |
| v228 | 0.028354 |
| v025 | 0.022844 |
| v463a | 0.016126 |
| v481a | 0.01335 |
| v463c | 0.005739 |
